# Development and Characterization of a Reverse Genetics System for a Human-Derived Severe Fever With Thrombocytopenia Syndrome Virus Isolate From South Korea

**DOI:** 10.3389/fmicb.2021.772802

**Published:** 2021-11-11

**Authors:** Seok-Min Yun, Tae-Young Lee, Hee-Young Lim, Jungsang Ryou, Joo-Yeon Lee, Young-Eui Kim

**Affiliations:** ^1^Division of Acute Viral Diseases, Center for Emerging Virus Research, National Institute of Infectious Diseases, National Institute of Health, Korea Disease Control and Prevention Agency, Cheongju-si, South Korea; ^2^Division of Emerging Virus and Vector Research, Center for Emerging Virus Research, National Institute of Infectious Diseases, National Institute of Health, Korea Disease Control and Prevention Agency, Cheongju-si, South Korea; ^3^Center for Emerging Virus Research, National Institute of Infectious Diseases, National Institute of Health, Korea Disease Control and Prevention Agency, Cheongju-si, South Korea

**Keywords:** severe fever with thrombocytopenia syndrome virus, reverse genetics system, SFTSV, animal model, recombinant virus

## Abstract

Severe fever with thrombocytopenia syndrome virus (SFTSV) is an emerging, tick-borne *Bandavirus* that causes lethal disease in humans. As there are no licensed vaccines and therapeutics for SFTSV, there is an urgent need to develop countermeasures against it. In this respect, a reverse genetics (RG) system is a powerful tool to help achieve this goal. Herein, we established a T7 RNA polymerase-driven RG system to rescue infectious clones of a Korean SFTSV human isolate entirely from complementary DNA (cDNA). To establish this system, we cloned cDNAs encoding the three antigenomic segments into transcription vectors, with each segment transcribed under the control of the T7 promoter and the hepatitis delta virus ribozyme (HdvRz) sequences. We also constructed two helper plasmids expressing the nucleoprotein (NP) or viral RNA-dependent RNA polymerase (RdRp) under the control of the T7 promoter and the encephalomyocarditis virus (EMCV) internal ribosome entry site (IRES). After co-transfection into BHK/T7-9 cells with three transcription and two helper plasmids, then passaging in Vero E6 or Huh-7 cells, we confirmed efficient rescue of the recombinant SFTSV. By evaluating the *in vitro* and *in vivo* virological properties of the parental and rescued SFTSVs, we show that the rescued virus exhibited biological properties similar to those of the parental virus. This system will be useful for identifying molecular viral determinants of SFTSV infection and pathogenesis and for facilitating the development of vaccine and antiviral approaches.

## Introduction

Severe fever with thrombocytopenia syndrome (SFTS), an emerging tick-borne viral infectious disease with a high fatality rate and symptoms including high fever, gastrointestinal symptoms, thrombocytopenia, and leukopenia, was first reported in China in 2009 (Yu et al., 2011) and was subsequently identified in South Korea and Japan in 2013 (Kim et al., 2013; Takahashi et al., 2014). Following the first report of SFTS in humans (Yu et al., 2011), the number of human cases has rapidly increased each year in East Asian countries. Unfortunately, currently there are no licensed vaccines or therapeutics for the prevention or treatment of severe fever with thrombocytopenia syndrome virus (SFTSV) infection.

Severe fever with thrombocytopenia syndrome virus, which has been renamed *Dabie bandavirus* belongs to the genus *Bandavirus* in the family *Phenuiviridae* of the order *Bunyavirales* (Kuhn et al., 2020). It has a tripartite RNA genome consisting of three negative-stranded RNA, designated large (L), medium (M), and small (S), which encode the RNA-dependent RNA polymerase (RdRp); the viral envelope glycoproteins (Gn and Gc); and a nucleocapsid protein (N) and a non-structural protein (NSs), respectively, in an ambisense orientation (Yu et al., 2011; Liu et al., 2014). The N and L proteins, together with the viral RNA, constitute a ribonucleoprotein (RNP) complex that is essential for genome replication and transcription (Zhou et al., 2013). It has been reported that SFTSV is transmitted via tick bites (Xu et al., 2011; Yu et al., 2011; Ding et al., 2014) and human-to-human transmission through close contact with the blood or body secretions of the infected patients (Bao et al., 2011; Gai et al., 2012; Liu et al., 2012; Chen et al., 2013; Tang et al., 2013; Kim et al., 2015).

The absence of vaccines and antiviral therapies against SFTSV emphasizes the need to focus on the molecular biology of SFTSV and its interaction with the host. In this respect, the reverse genetics (RG) system, which enables the generation of infectious virus from cloned complementary DNA (cDNA), is an effective tool for understanding the mechanisms of pathogenesis and the development of vaccines and therapeutics. To date, several groups have reported RG systems for the recovery of viruses belonging to the family *Phenuiviridae*, including Rift Valley fever virus (Ikegami et al., 2006; Gerrard et al., 2007; Billecocq et al., 2008; Habjan et al., 2008) and SFTSV (Brennan et al., 2015) using the T7 RNA polymerase or RNA polymerase I.

In the present study, we developed an RG system for the clinical SFTSV isolate KASJH from a Korean patient identified in 2014. We have characterized it using infectivity and pathogenicity tests *in vitro* and *in vivo*, as well as by genome sequencing. This system provides a platform for elucidating the molecular mechanisms involved in host tropism and pathogenesis of SFTSV infection.

## Materials and Methods

### Ethics Statement

All animal experiments were conducted in an enhanced biosafety level 3 (BSL3) laboratory in conformance with the guidelines of the Institutional Animal Care and Use Committee (IACUC) of the Korea Centers for Disease Control and Prevention (approval number, KCDC-073- 19-2A).

### Cell Lines and Virus

BHK/T7-9 (RIKEN BRC cell bank, RCB4942, Ibaraki, Japan), a cell line derived from BHK-21 that stably expresses T7 RNA polymerase (Ito et al., 2003), was cultured in Dulbecco’s modified Eagle’s Medium (DMEM; Gibco) supplemented with 10% fetal bovine serum (FBS; Gibco), 10% tryptose phosphate broth (TPB; BD), and 1% penicillin/streptomycin at 37°C with 5% CO_2_. Vero E6 and Huh-7 cells were maintained in DMEM supplemented with 10% FBS and 1% antibiotics. The SFTSV strain KASJH was isolated from a Korean SFTS patient’s serum in 2014 (Yun et al., 2015).

### Construction of Plasmids

Plasmids required for the rescue of SFTSV have been described previously (Brennan et al., 2015). We were kindly provided pTVT7-HB29L and pTM1-HB29L plasmids by Benjamin Brennan from MRC-University of Glasgow Centre for Virus Research. Using these plasmids as templates, we constructed plasmids containing the sequences of the Korean KASJH strain (GenBank accession nos. KP663731 to KP663733) on behalf of the Chinese HB29 strain using synthetic gene constructs and In-fusion cloning strategy. Plasmids containing full-length S, M, and L segments of KASJH strain in antigenomic sense flanked by the T7 promoter and hepatitis delta virus ribozyme (HdvRz) sequence were designated as pTVT7_KASJH_L(+), pTVT7_KASJH_M(+), and pTVT7_KASJH_S(+), respectively. Helper plasmids pTM1_KASJH_NP and pTM1_KASJH_RdRp contain the L and N open reading frames (ORFs) of KASJH under the control of the T7 promoter and encephalomyocarditis virus (EMCV) internal ribosome entry site (IRES) sequence. All plasmids were sequenced to ensure no undesired mutations. Primers used for the construction of these plasmids are available upon request.

### Generation of Recombinant Severe Fever With Thrombocytopenia Syndrome Virus From Complementary DNA and Sequence Analysis

**FIGURE 1:**
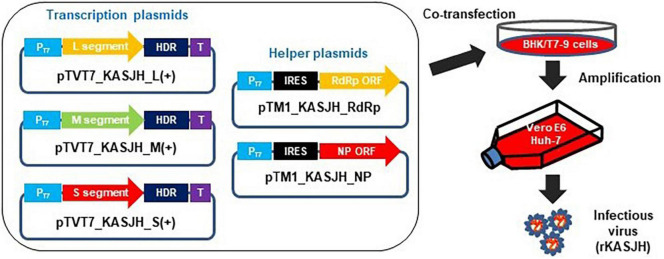
Schematic describing the generation of recombinant SFTSV using a reverse genetics system. BHK/T7-9 cells were co-transfected with three transcription and two helper plasmids. rKASJH was rescued from the culture supernatants of transfected BHK/T7-9 cells, followed by passages on Vero E6 or Huh-7 cells.

Subconfluent BHK/T7-9 cells (1 × 10^6^ per 100-mm dish) were transfected using 3 μL Lipofectamine-3000 (Invitrogen) per ug of DNA in 500 μL Opti-MEM (Gibco), with 2 μg each of pTVT7_KASJH_L(+), pTVT7_KASJH_M(+), pTVT7_KASJH_S(+), 1 μg pTM1_KASJH_NP, and 0.2 μg pTM1_KASJH_RdRp. To distinguish between recombinant and parental viruses, we used an empty vector as a helper plasmid instead of one containing the KASJH RdRp gene. After 6 days incubation at 37°C, supernatants were collected, clarified, and passaged three times in Vero E6 or Huh-7 cells. Supernatants were harvested at 15 days post-infection (dpi). The rescued virus was designated rKASJH to distinguish it from the parental KASJH (wtKASJH) (Figure 1). To identify sequence differences between parental and rescued viruses, we sequenced the ORFs of these viruses using the Sanger method and DNAStar software (version 5.0.6) as previously described (Yun et al., 2017).

### Growth Kinetics and Viral Protein Synthesis in Cell Culture

Subconfluent monolayer cultures of Vero E6 and Huh-7 cells growing in 24-well plates were infected with parental and rescued viruses at a multiplicity of infection (MOI) of 0.01 or 1. Cells were incubated at 37°C in 5% CO_2_. Supernatants were harvested daily for 7 days and stored in aliquots at −70°C prior to titration. The titers of SFTSV in daily samples were determined by focus formation on Vero E6 cells. Cells were also harvested from the daily samples described above for growth kinetics. Cell lysates were prepared and subjected to western blotting at the same time points to quantify the expression of viral N and NSs proteins.

### Virus Titration

Vero E6 cells (3 × 10^5^ per well) were seeded in 6-well plates and infected with 10-fold serial dilutions of virus for 1 h at 37°C, followed by the addition of 5 mL/well of overlay medium containing 2× DMEM (Welgene, Daegu, South Korea), 10% FBS, 1% penicillin/streptomycin, and 0.5% SeaKem LE Agarose (Lonza). Cells were incubated at 37°C for 7 days and then fixed with 10% formaldehyde in PBS. After washing with PBS, cells were permeabilized in 10% Triton X-100 in PBS for 10 min. Anti-SFTSV nucleoprotein (NP) monoclonal antibody (6B3, in-house) and HRP-conjugated anti-mouse IgG antibody were used for immunostaining the infected cells. Foci were detected using 3,3′-diaminobenzidine substrate (Vector Laboratories). Visible foci were counted and used to calculate viral titers.

### Western Blotting

At different time points after infection, cell lysates were prepared by the addition of NP40 lysis buffer (Invitrogen) and protease inhibitor (Roche). Equal aliquots of lysates were heat denatured and separated on a 4–12% SDS-PAGE gel (Invitrogen). Separated proteins were transferred to a methanol-activated polyvinylidine difluoride (PVDF) membrane (Bio-Rad) and blocked in PBS containing 5% skim milk and 0.1% Tween 20. The blot was incubated with mouse anti-N (6B3, in-house), anti-NSs (8G4, in-house), and rabbit anti-GAPDH (Santa Cruz Biotechnology) monoclonal antibodies. The antigen-primary antibody complexes were recognized by alkaline phosphatase (AP)-conjugated goat anti-mouse or anti-rabbit IgG (Jackson ImmunoResearch) antibodies, as appropriate. Visualization of detected proteins was performed by leveraging the colorimetric detection of AP activity using 5-bromo-4-chloro-3-indolyl phosphate (BCIP) and nitro blue tetrazolium (NBT) substrate.

### Pathogenicity in Mice

Mice lacking the type 1 interferon receptor (C57BL/6 IFNAR^–/–^) were purchased from the Jackson Laboratory. Briefly, 8 to 10-week-old male C57BL/6 IFNAR^–/–^ mice (*n* = 5 per group) were infected with high and low (1 × 10^5^ and 1 × 10^2^ FFU, respectively) doses of SFTSV (100 μL) via the intramuscular (IM) route. DMEM was used to dilute the virus to the desired concentration. Control mock-infected mice were inoculated with DMEM by the same route. Body weight and clinical symptoms were observed for 14 days after inoculation; mice with a weight loss of more than 20% were euthanized. Spleen, liver, kidney, and serum samples were collected at 1, 3, and 5 dpi. Organs were individually weighed, homogenized, and prepared as 10% suspensions (w/v) in PBS containing 10% FBS. Suspensions were clarified by centrifugation (2,000 rpm for 5 min, 4°C) and stored at −70°C prior to titration. For virus titration in sera and the organ suspensions, focus-forming assays on Vero E6 cells were performed as described above. Viral RNA copy number was measured as described below.

### Real-Time Quantitative Reverse Transcription Polymerase Chain Reaction

Total RNA was extracted using the QIAamp viral RNA Mini kit (Qiagen) according to the manufacturer’s protocol. Viral copy numbers were determined by real-time Reverse transcription polymerase chain reaction (qRT-PCR) with an M segment-based SFTSV-specific primer set. The forward primer was 5′-GTCATCATCATATTTTGTTCCTGATGC-3′ and the reverse primer was 5′-GGGCACCCAGACTGGCAG TC-3′. The probes were JOE/5′-TCCAGGTGTACATCAGTGAG GAGATGTCG-3′/BHQ and JOE/5′-TCCAGGTGTACATCTGT GAGGAGATGTCG-3′/BHQ. Copy numbers were calculated as ratios normalized to the control by a standard curve method. Real-time RT-PCRs were performed using a PowerChek SFTSV Real-time PCR kit (Kogenebiotech, Seoul, South Korea) on a 7500 Real-time RT-PCR system (Applied Biosystems).

### Histopathological Analysis

Eight-to-ten-week-old male C57BL/6 IFNAR^–/–^ mice in groups of three were inoculated IM with 100 μL of 10^2^ FFU of either parental or rescued SFTSVs, whereas three control mice were mock inoculated with DMEM. All mice were observed daily and euthanized on day 1, 3, and 5 post-infection. Spleen, liver, and kidney tissues were separately harvested and fixed in 4% paraformaldehyde for 1 day at room temperature. For histopathology, fixed tissues were bisected, embedded in paraffin, sectioned at 3 μm, and stained with hematoxylin and eosin (H&E).

### Statistical Analyses

All analyses were performed using GraphPad Prism version 7.0 (GraphPad Software Inc.). All data are presented as the mean ± s.d. (standard deviation). The *in vitro* growth kinetics were evaluated using an unpaired, two-tailed *t*-test. Survival was estimated using the Kaplan-Meier method and analyzed by log-rank analysis. For the analyses of viral load in serum and tissues, two-way analysis of variance (ANOVA) with Tukey’s test was used. *P*-values less than 0.05 were considered statistically significant.

## Results

### Recovery of Recombinant Severe Fever With Thrombocytopenia Syndrome Virus From Full-Length Complementary DNA Clones

We used a human-derived SFTSV isolate to construct the cDNA clone. This strain (KASJH) was isolated from a 69-year-old South Korean patient in 2014. As mentioned above, three transcription and two helper plasmids containing the viral sequences (GenBank accession Nos. KP667331-KP667333) were constructed using synthetic gene constructs and an In-Fusion cloning strategy. Sequences of the inserted viral genome were confirmed by sequencing. To recover rKASJH from full-length cDNA clones, three transcription (pTVT7_KASJH_L(+), pTVT7_KASJH_M(+), and pTVT7_KASJH_S(+)) and two helper plasmids (pTM1_KASJH_NP and pTM1_KASJH_RdRp) were co-transfected into BHK/T7-9 cells and passaged in Vero E6 or Huh-7 cells. We confirmed that the recombinant SFTSV was efficiently rescued by real-time qRT-PCR and focus-forming assays. We confirmed that the recombinant SFTSV was efficiently rescued by real-time qRT-PCR and focus-forming assays. The rescued viruses from passages in Huh-7 and Vero E6 cells reached a titer of 5.2 × 10^6^ and 4.5 × 10^5^ FFU/mL, respectively. The results revealed that the rescued virus from passages in Huh-7 cells replicated more effectively than in Vero E6 cells. No virus was detected in controls in which the helper plasmid encoding the RdRp sequence of KASJH was excluded. These results demonstrate that the recombinant virus was produced from cloned cDNAs following co-transfection and passage. As summarized in Table 1, comparison of the recombinant and parental virus sequences revealed two single-base mutations in the L segment, one of which was translationally silent.

**TABLE 1:** Sequence differences between the parental and recombinant SFTSVs.

**Segment**	**Position^a^**		**Nucleotide changes**		**Amino acid changes**	
	**Nucleotide**	**Residue**	**Parental (wtKASJH)**	**Recombinant (rKASJH)**	**Parental (wtKASJH)**	**Recombinant (rKASJH)**
L	4	2	A	G	N	D
	3,741	1,247	C	T	Y	Y^b^

*^a^Nucleotide and amino-acid residue numbers are based on the complete ORF sequence of the KASJH strain, GenBank accession number KP663731.*

*^b^Silent mutation.*

### *In vitro* Growth Properties of Parental Isolate and Rescued Virus

**FIGURE 2:**
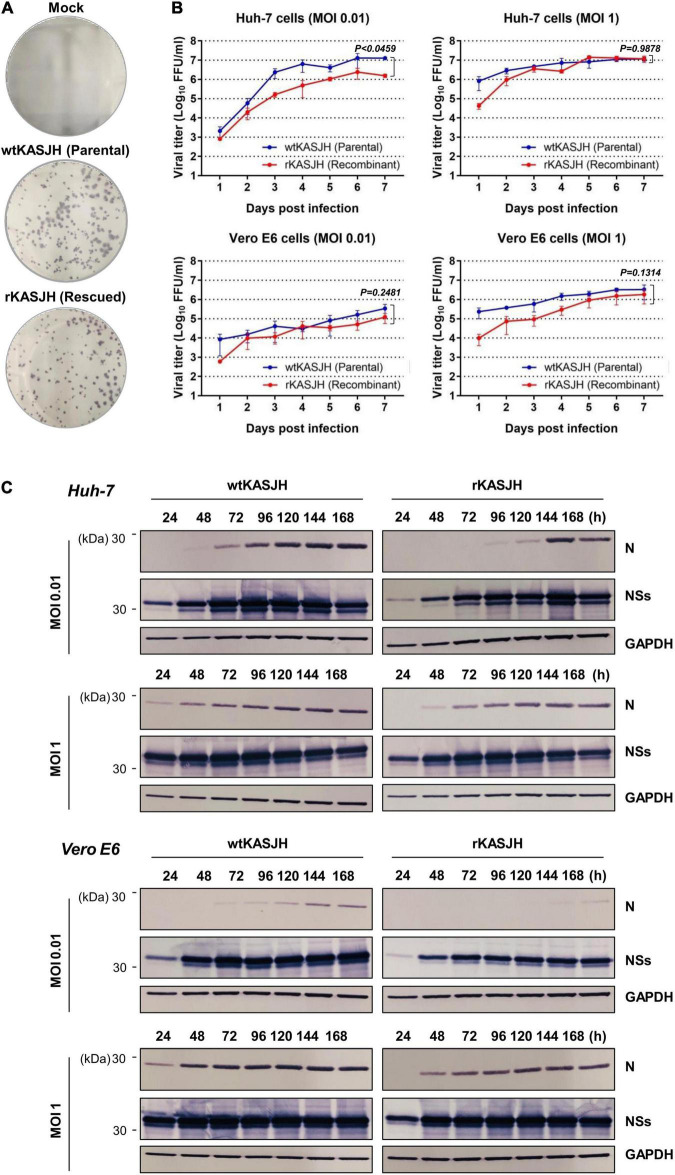
Characterization of parental and rescued SFTSVs in cell culture. **(A)** Representative focus morphology in Vero E6 cells immunostained with anti-SFTSV N antibody 7 dpi. **(B)** Growth kinetics of Vero E6 and Huh-7 cells infected with wtKASJH or rKASJH at MOIs of 0.01 and 1 FFU/cell and infections were performed in triplicate. Culture supernatants were collected from the infected cells at the indicated days after infection and virus titers were determined by focus-forming assays. Error bars indicate standard deviations of the mean. Statistical significance between wtKASJH and rKASJH infected groups was determined by an unpaired, two-tailed *t*-test. **(C)** Viral protein synthesis from parental and recombinant viruses in Vero E6 and Huh-7 cell lysates detected by immunoblotting with anti-SFTSV N, anti-SFTSV NSs, and anti-GAPDH antibodies.

To analyze the *in vitro* properties of the recombinant SFTSV, we first examined plaque phenotypes and growth kinetics of the rKASJH compared with the parental KASJH strain in susceptible Vero E6 and Huh-7 cells over 7 dpi at MOIs of 0.01 and 1. As shown in Figure 2A, there was no obvious difference in the morphology of foci formed by the parental and recombinant SFTSVs. Comparing replication kinetics of parental and recombinant viruses in Vero E6 and Huh-7 cells, both cells infected with parental and rescued SFTSVs at MOIs of 0.01 and 1 exhibited cytopathic effects (CPE) on 3 to 7 dpi and rKASJH grew nearly as efficiently as wtKASJH, with minor differences in kinetics and maximum viral titers varying by cell type and MOI (Figure 2B). In Huh-7 cells infected with SFTSVs at an MOI of 1, the titer of rKASJH was >10-fold lower than that of wtKASJH at 1 dpi, comparable to that of wtKASJH at 2–7 dpi. Similar growth kinetics were also observed in Vero E6 cells. The peak titers in Huh-7 cells infected with wtKASJH and rKASJH at both MOIs were higher than those in Vero E6 cells.

To compare the viral protein expression profiles of wtKASJH and rKASJH, we measured the expression levels of viral N and NSs proteins produced in Vero E6 and Huh-7 cells at 1–7 dpi at MOIs of 0.01 and 1. Immunoblotting with mouse mAbs specific for SFTSV N and NSs revealed similar expression levels between viruses (Figure 2C).

Collectively, these results demonstrate that the recombinant SFTSV exhibited similar biological properties *in vitro* to those of the parental virus.

### Pathogenesis of Parental Isolate and Rescued Virus in Mice

**FIGURE 3:**
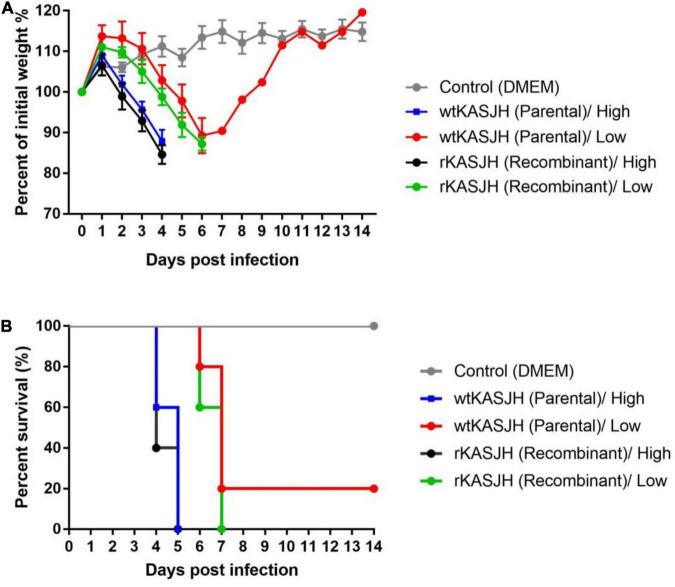
Virulence of parental and rescued SFTSVs in C57BL/6 IFNAR^–/–^ mice. Groups of five mice were inoculated intramuscularly with high (1 × 10^5^ FFU) and low (1 × 10^2^ FFU) doses of the indicated viruses and then monitored for weight loss **(A)** and mortality rate **(B)**. Error bars indicate standard deviations of the mean. Survival was quantified using the Kaplan-Meier method and tested for significance by log-rank analysis. ns, not significant.

To compare the *in vivo* virulence of the parental and recombinant SFTSVs, we used 8 to 10-week-old C57BL/6 IFNAR^–/–^ mice, a well-established immunodeficient mouse model for SFTSV infection. Groups of five mice were inoculated intramuscularly with high (10^5^ FFU) and low (10^2^ FFU) doses of wtKASJH and rKASJH. Compared with mock-infected mice, mice infected with the high dose of both the parental and rescued SFTSVs lost weight starting at 2–3 dpi. Mortality was observed starting on 4 dpi for both wtKASJH and rKASJH, with all mice dead at 5 dpi (Figures 3A,B). In mice infected with the low dose, both groups lost weight starting at 4–5 dpi. Except for one mouse infected with the low dose of wtKASJH, mortality was observed starting on 6 dpi for both the two viruses, with all mice dead at 7 dpi (Figures 3A,B). The log-rank test indicated that there was no significant difference in survival between groups (Figure 3B).

**FIGURE 4:**
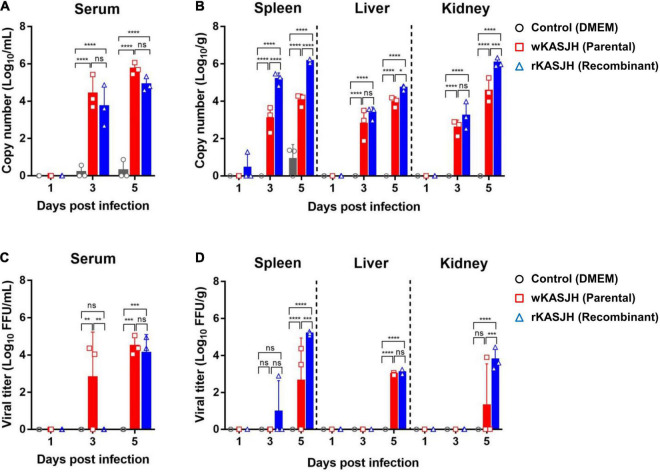
Viral RNA copy numbers and titers in serum **(A,C)** and tissues **(B,D)** from C57BL/6 IFNAR^–/–^ mice infected with parental and recombinant SFTSVs. Samples were collected at 1, 3, and 5 dpi. Copy numbers and titers were determined by real-time qRT-PCR and focus-forming assays, respectively. Data are represented as the mean ± s.d. Significance was determined by 2-way ANOVA with Tukey’s multiple comparisons test. *p < 0.05; **p < 0.01; ***p < 0.001; ****p < 0.0001.

To examine the viral tissue distribution and serum, tissue and serum samples were collected from 8 to 10-week-old male C57BL/6 IFNAR^–/–^ mice in groups of three on 1, 3, and 5 dpi. Using the real-time qRT-PCR method, both wtKASJH and rKASJH were detected at 3 and 5 dpi in organs (spleen, liver, and kidney) and serum samples of the infected C57BL/6 IFNAR^–/–^ mice (Figures 4A,B). Serum viral RNA copy number did not differ between wtKASJH and rKASJH-infected groups (Figure 4A). There was, however, a significant difference in copy number in tissues (spleen, liver, and kidney) at 5 dpi (Figure 4B). Remarkably, copy numbers were the highest in the serum, spleen, and kidney at 5 dpi. The viral titers in serum samples and tissues quantified by focus-forming assays revealed that viral load peaked at 5 dpi in the serum, spleen, liver, and kidney of both wtKASJH- and rKASJH-infected groups (Figures 4C,D). Among the organs used in this study, the spleen was the most susceptible organ, as it showed the highest viral titer (5.2log_10_FFU/g) 5 dpi after rKASJH infection.

**FIGURE 5:**
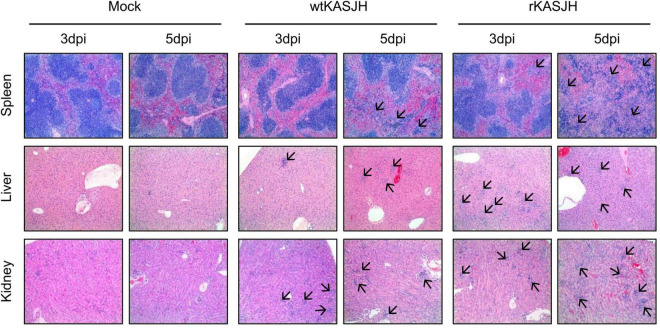
Histopathological analysis of mice infected with parental and rescued SFTSVs. Tissues were harvested at 3 and 5 dpi and processed for H&E staining. Both viruses caused severe lesions that were absent in tissues from mock-infected control mice. Arrows indicate inflamed regions. Magnification, 100×.

Next, to investigate viral pathology, multiple tissues of C57BL/6 IFNAR^–/–^ mice infected with mock, wtKASJH, and rKASJH were examined by staining organ sections with H&E. As shown in Figure 5, we found that tissues from mice infected with both viruses exhibited cell infiltration, necrotizing hepatitis, and lymphoid depletion that was not observed in the mock-infected group. Moreover, more severe histopathological changes were observed at 5 dpi than at 3 dpi. In agreement with the viral load data, the most severe lesions were identified at 5 dpi in the spleens of rKASJH-infected mice.

Together, our results showed that the rescued virus has similar *in vivo* characteristics as the parental virus on pathogenicity in C57BL/6 IFNAR^–/–^ mice.

## Discussion

Severe fever with thrombocytopenia syndrome is an emerging infectious disease caused by SFTSV infection and has high mortality and increasing prevalence in East Asia. However, there are currently no licensed vaccines or antivirals for controlling this virus. An RG system is a powerful tool for studying the virus life cycle and pathogenesis, with additional utility for development of vaccines and therapeutics against negative-strand RNA viruses (Bridgen and Elliott, 1996; Palese et al., 1996; Bouloy and Flick, 2009; Hoenen et al., 2011). Among them, development of an RG system for SFTSV was first reported for the Chinese HB29 strain in 2015 (Brennan et al., 2015). This system utilized BSR-T7/5 cells stably expressing T7 RNA polymerase to rescue the recombinant SFTSVs.

In this study, we report the development of a T7 RNA polymerase-driven plasmid-based RG system for a clinical Korean isolate, KASJH, based on the plasmid backbones used in the first reported RG system. We have used this system to rescue a recombinant SFTSV. This recombinant virus, rKASJH, was indistinguishable from the parental KASJH strain with respect to patterns of viral protein synthesis and growth kinetics in cultured mammalian cells, as well as virulence in mice.

We first investigated the *in vitro* phenotypic properties of the parental and rescued SFTSVs by examining focus formation and growth curves and viral protein expression profiles on Vero E6 and Huh-7 cells. Compared with the parental virus, we found that the foci phenotypes produced by the rescued virus on Vero E6 cells showed a similar morphology and the replication efficiency of the rescued virus was represented similar kinetics in Vero E6 and Huh-7 cells (Figures 2A,B). According to the dpi with each virus, the expression level of viral N and NSs proteins were detected by immunoblotting and there were no discernible differences in the patterns of expression profiles (Figure 2C). These results showed that the *in vitro* biological properties of the rescued virus resembled those of the parental virus.

Experimental assessment of the pathogenicity in animal models is an important aspect of the development of vaccine and antiviral therapies. We therefore evaluated the virulence of the parental and rescued SFTSVs in a previously described mouse model of SFTSV infection (Tani et al., 2016, 2018; Matsuno et al., 2017).

For *in vivo* experiments, although the tissue distributions and viremia following infection by the parental and rescued SFTSVs showed significant minor differences, there was no significant difference in survival between them. Additionally, histopathological analysis revealed lesions in the tissues of wtKASJH or rKASJH-infected groups (Figure 5). These data indicate that the rescued virus displayed a pathogenesis in C57BL/6 IFNAR^–/–^ mice similar to that observed in case of the parental virus.

Sequencing revealed a single amino-acid substitution (Asn to Asp) at position 2 in the L segment of rescued virus that was not present in the parental virus (Table 1). As we observed no differences in *in vitro* and *in vivo* characteristics between the parental and rescued SFTSVs, this amino-acid residue identified in this study did not greatly affect viral replication and virulence.

In summary, we have established a T7 RNA polymerase-driven RG system for production and genetic manipulation of infectious SFTSV isolated from South Korea. The system reported here will be a valuable tool for studying the molecular biology of SFTSV, viral determinants of SFTSV pathogenesis, and the development of vaccines and antivirals against SFTSV infection. Further study is warranted using site-directed mutagenesis to identify pathogenic determinants of SFTSV and their underlying mechanisms.

## Data Availability Statement

The original contributions presented in the study are included in the article/supplementary material, further inquiries can be directed to the corresponding author/s.

## Ethics Statement

The animal study was reviewed and approved by Institutional Animal Care and Use Committee (IACUC) of the Korea Centers for Disease Control and Prevention (approval number, KCDC-073-19-2A).

## Author Contributions

S-MY and Y-EK conceived the study, designed the experiments, analyzed and interpreted the data, and wrote the manuscript. S-MY, T-YL, and Y-EK performed the experiments and contributed to materials. H-YL, JR, and J-YL reviewed the manuscript. Y-EK supervised the study. All authors approved the final version of the manuscript.

## Conflict of Interest

The authors declare that the research was conducted in the absence of any commercial or financial relationships that could be construed as a potential conflict of interest.

## Publisher’s Note

All claims expressed in this article are solely those of the authors and do not necessarily represent those of their affiliated organizations, or those of the publisher, the editors and the reviewers. Any product that may be evaluated in this article, or claim that may be made by its manufacturer, is not guaranteed or endorsed by the publisher.
